# Relationships Between Various Parameters of Prolonged Sedentary Bouts and Health-Related Quality of Life (HRQOL) in Patients on Chronic Hemodialysis: A Cross-Sectional Study

**DOI:** 10.7759/cureus.70126

**Published:** 2024-09-24

**Authors:** Kentaro Sugahara, Nobuyuki Miyatake, Takashi Kondo, Keiichi Namio, Shuhei Hishii, Hiroyuki Nishi, Kazuhiro Ujike, Kiichi Koumoto, Hiromi Suzuki, Yorimasa Yamamoto

**Affiliations:** 1 Hygiene, Kagawa University, Miki, JPN; 2 Physical Therapy, Vocational School Anabuki Rehabilitation College, Takamatsu, JPN; 3 Clinical Engineering, Innoshima General Hospital, Onomichi, JPN; 4 Epidemiology and Public Health, Kyushu Medical Sports Vocational School, Kitakyushu, JPN; 5 Internal Medicine, Innoshima General Hospital, Onomichi, JPN

**Keywords:** chronic hemodialysis, eq-5d, health-related quality of life (hrqol), non-hemodialysis days, prolonged sedentary bouts

## Abstract

Background: Previous studies have revealed that sedentary behavior, including quantitative and qualitative parameters, was associated with health-related quality of life (HRQOL) in patients on chronic hemodialysis.

Objective: The present study examined the relationships between various parameters of prolonged sedentary bouts on non-hemodialysis days and HRQOL in patients on chronic hemodialysis.

Methods: A total of 110 patients (47.2%) on chronic hemodialysis (64 men and 46 women, aged 71.4 ± 11.2 years) among 233 patients, who were outpatients at a hospital in Hiroshima prefecture, Japan, were enrolled in this cross-sectional study. Prolonged sedentary bouts were evaluated using a tri-accelerometer, i.e., sedentary behavior (minutes), median sedentary bouts (minutes), maximum sedentary bouts (minutes), sedentary bouts (bouts and minutes/bout), and prolonged sedentary bouts (≥5, ≥10, ≥30, and ≥60 minutes; bouts and minutes/bout). HRQOL was assessed using EuroQol 5-Dimension (EQ-5D).

Results: The EQ-5D score was 0.755 ± 0.216. All prolonged sedentary parameters, except for sedentary bouts (bouts), correlated with HRQOL, and higher correlation coefficients were observed between sedentary behavior (minutes) and HRQOL (r = -0.416) and between maximum sedentary bouts (minutes) and HRQOL (r = -0.436) than between other parameters. These parameters were also identified as factors affecting HRQOL, even after adjustments for confounding factors.

Conclusion: In summary, sedentary behavior (minutes) and maximum sedentary bouts (minutes) on non-hemodialysis days, which may be representative of quantitative parameters for sedentary behavior, correlated with HRQOL in patients on chronic hemodialysis.

## Introduction

The number of patients on chronic hemodialysis has markedly increased, and in 2023, the Japanese Society of Dialysis Therapy reported that 349,700 patients were on chronic hemodialysis [1]. Therefore, a proper strategy to reduce the number of patients on chronic hemodialysis and improve their daily life is urgently required and has become a public health challenge in Japan.

Sedentary behavior, which is defined as energy expenditure <1.5 metabolic equivalents (METs) while in a sitting or reclining posture [2], is a critical factor involved in various health outcomes, such as obesity and type 2 diabetes mellitus [3-5], and longer sedentary behavior is closely associated with mortality [6-8]. We previously reported that sedentary behavior (%), which may be representative of quantitative parameters for sedentary behavior, correlated with health-related quality of life (HRQOL) in a cross-sectional study [9] and mortality in a longitudinal study [10]. Prolonged sedentary bouts (≥30 and ≥60 minutes; minutes, bout, and %), which may be representative of qualitative parameters for sedentary behavior, were also related to HRQOL in a cross-sectional study [11]. In addition, the parameters of prolonged sedentary bouts on non-hemodialysis days were identified as factors affecting mortality in a survival analysis [12]. However, the relationships between various parameters of prolonged sedentary bouts and HRQOL remain unclear; therefore, a more detailed understanding of the parameters that have an impact on HRQOL will provide useful information for improving the daily life of patients on chronic hemodialysis. Based on these findings, the aim of this present study was to examine the relationships between various parameters of prolonged sedentary bouts on non-hemodialysis days and HRQOL in patients on chronic hemodialysis.

## Materials and methods

Subjects

**Figure 1:**
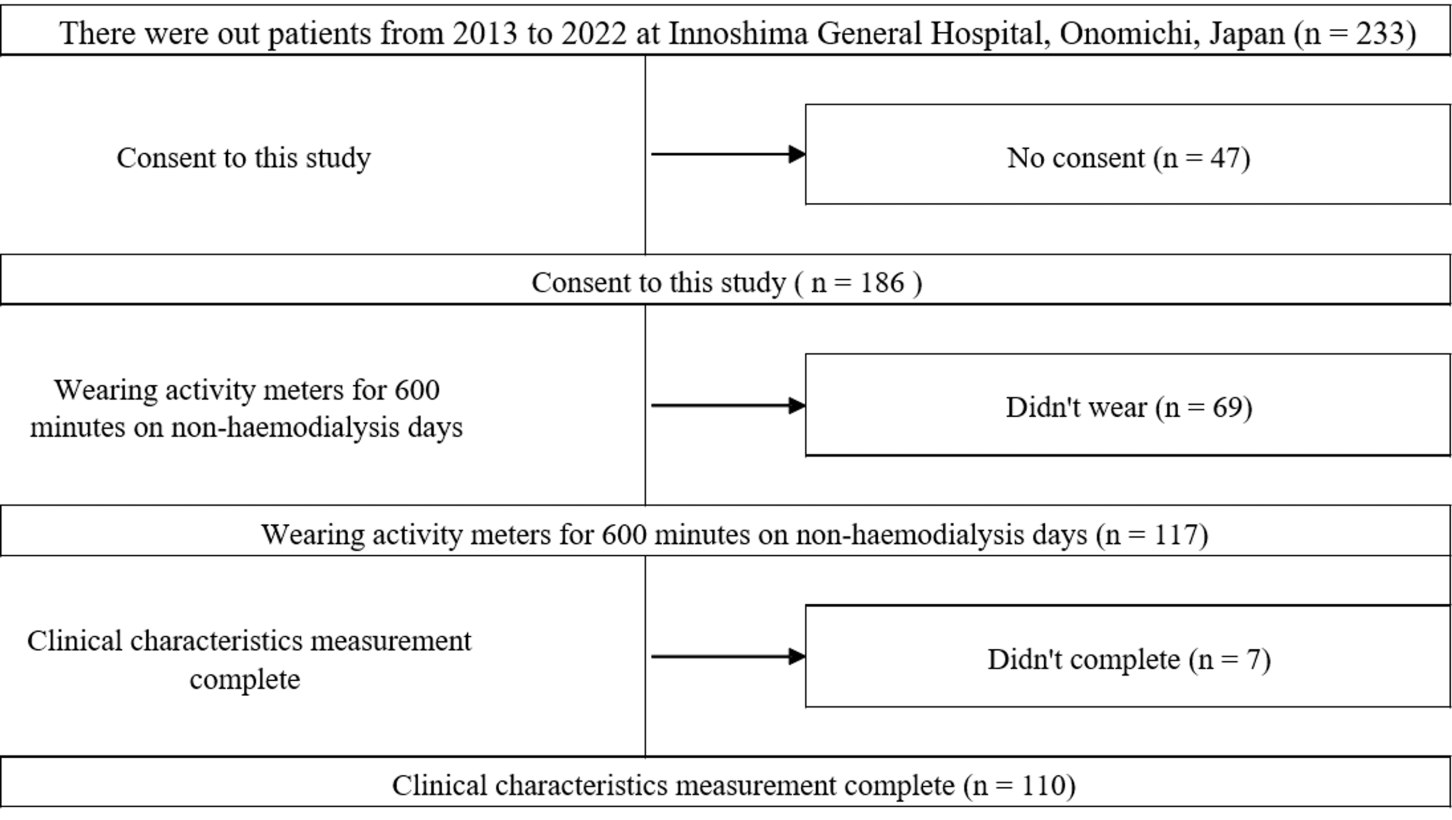
Flow diagram of analysis

A total of 110 outpatients (47.2%) (64 men and 46 women, aged 71.4 ± 11.2 years) on chronic hemodialysis out of 233 patients who met the following criteria were enrolled in this cross-sectional study: (1) recruited between September 2013 and September 2022 at a hospital in Onomichi, Japan; (2) underwent measurements for parameters of prolonged sedentary bouts and HRQOL; and (3) provided written informed consent (Figure 1).

Ethical approval was obtained from the Ethical Committee of Innoshima General Hospital, Onomichi, Japan (Approval number (date): H25-2-27 (Feb 27, 2013), H26-1-23 (Jan 23, 2014), H26-12-16 (Dec 16, 2014), H27-12-25 (Dec 25, 2015), H28-12-9 (Dec 9, 2016), H29-12-4 (Dec 4, 2017), H30-11-1 (Nov 11, 2018), H31-2-25 (Feb 25, 2019), R1-12-23 (Dec 23, 2019), R2-11-9 (Nov 9, 2020), R3-11-10 (Nov 10, 2021), R6-4-22 (Apr 22, 2024)).

Prolonged sedentary bouts

We evaluated prolonged sedentary bouts using a tri-accelerometer named Active Style Pro HJA 350-IT® (Omron, Kyoto, Japan), which was described in a previous study [13]. Subjects were asked to continuously wear the tri-accelerometer, except when sleeping or bathing. The not wear time was defined as a 0 physical activity level (metabolic equivalents (METs)) that continued for ≥60 minutes, while the wear time was defined as 24 hours (1440 minutes) - the not wear time. We assessed various parameters of prolonged sedentary bouts, i.e., sedentary behavior (minutes), median sedentary bouts (minutes), maximum sedentary bouts (minutes), sedentary bouts (bouts and minutes/bout), and prolonged sedentary bouts (≥5, ≥10, ≥30, and ≥60 minutes; bouts and minutes/bout).

Health-related quality of life (HRQOL)

HRQOL was examined using the Japanese version of EuroQol 5-Dimension 3-Level (EQ-5D-3L) [14]. EQ-5D consists of five scales with three levels describing 3⁵ (243 possible) possible health combinations. The five scales were as follows: (1) mobility, (2) self-care, (3) usual activities, (4) pain/discomfort, and (5) anxiety/depression.

Clinical parameters

We also obtained a number of clinical parameters from medical records, including age, sex, height (cm), body weight (dry weight) (kg), body mass index (BMI: kg/m^2^), duration of hemodialysis (months), and history of diabetes mellitus (yes/no). BMI was calculated as body weight (kg)/(height (m^2^)). The following blood parameters were measured using conventional methods: albumin (mg/dL), blood glucose (mg/dL), triglycerides (mg/dL), and high-density lipoprotein (HDL) cholesterol (mg/dL).

Statistical analysis

Data were expressed as the mean ± standard deviation (SD) and the number of subjects (%). The relationships between various parameters of prolonged sedentary bouts and EQ-5D scores were examined using a simple correlation analysis. By using a multiple regression analysis, we further evaluated the relationships between these parameters and EQ-5D scores after adjustments for confounding factors, such as sex, the duration of hemodialysis, age, BMI, and albumin, which are clinically important. All statistical analyses were performed using JMP Pro 16 (SAS, Cary, NC, USA).

## Results

Table 1 shows the clinical profiles of chronic hemodialysis patients on non-hemodialysis days. Sedentary behavior, median sedentary bouts, and maximum sedentary bouts were 723.0 ± 255.6, 5.4 ± 5.0, and 132.5 ± 67.9 minutes, respectively. The EQ-5D score was 0.755 ± 0.216, and 34 patients (30.9%) on chronic hemodialysis had a history of diabetes mellitus.

**Table 1:** Clinical characteristics of patients on chronic hemodialysis SD: standard deviation, EQ-5D: EuroQol 5-Dimension, HDL: high-density lipoprotein.

	Mean ± SD			Minimum	Maximum
						All patients = 110					
Age (years)	71.4 ± 11.2			41.0	92.0
Height (cm)	156.3 ± 8.6			133.6	175.6
Body weight (dry weight) (kg)	54.6 ± 10.6			35.3	91.0
Body mass index (kg/m^2^)	22.3 ± 3.4			16.5	31.5
Duration of hemodialysis (months)	68.8 ± 79.3			2.0	338.0
EQ-5D scores	0.755 ± 0.216			-0.062	1.000
Blood sample					
Albumin (g/mL)	3.7 ± 0.4			2.4	5.2
Blood glucose (mg/dL)	132.3 ± 42.6			81.0	302.0
Triglyceride (mg/dL)	111.1 ± 66.4			29.0	431.0
HDL cholesterol (mg/dL)	54.3 ± 16.2			19.1	108.7
Physical activity (non-hemodialysis days)					
Wear time (min/day)	973.1 ± 216.3			657.8	1436.8
Sedentary behavior (min)	723.0 ± 255.6			304.5	1312.5
Prolonged sedentary bouts (≥5 min) (bouts)	653.8 ± 254.5			233.5	1225.8
Prolonged sedentary bouts (≥10 min) (min)	595.5 ± 261.0			163.3	1179.8
Prolonged sedentary bouts (≥30 min) (min)	426.4 ± 253.1			43.8	1062.5
Prolonged sedentary bouts (≥60 min) (min)	273.9 ± 218.2			0.0	989.3
Median sedentary bouts (min)	5.4 ± 5.0			1.4	31.6
Maximum sedentary bouts (min)	132.5 ± 67.9			33.5	312.0
Sedentary bouts (bouts)	59.7 ± 18.0			19.8	115.3
Prolonged sedentary bouts (≥5 min) (bouts)	25.2 ± 6.8			11.5	43.5
Prolonged sedentary bouts (≥10 min) (bouts)	16.4 ± 5.0			7.5	30.0
Prolonged sedentary bouts (≥30 min) (bouts)	6.2 ± 3.0			0.8	14.8
Prolonged sedentary bouts (≥60 min) (bouts)	2.5 ± 1.8			0.0	7.8
Sedentary bouts (min/bouts)	13.9 ± 8.9			4.6	52.7
Prolonged sedentary bouts (≥5 min) (min/bout)	27.0 ± 11.7			12.0	71.2
Prolonged sedentary bouts (≥10 min) (min/bout)	36.5 ± 13.6			19.2	91.6
Prolonged sedentary bouts (≥30 min) (min/bout)	65.8 ± 17.6			37.1	118.1
Prolonged sedentary bouts (≥60 min) (min/bout)	25.6 ± 18.0			0.0	79.1

We then investigated the relationships between various parameters of prolonged sedentary bouts and HRQOL using a simple correlation analysis (Table 2). These parameters, except for sedentary bout (bouts), correlated with EQ-5D scores. Sedentary behavior (minutes) (r = -0.416) and maximum sedentary bouts (minutes) (r = -0.436) correlated with HRQOL, while the other parameters did not.

**Table 2:** Simple correlation analysis between EQ-5D scores and prolonged sedentary bouts *p < 0.05. EQ-5D: EuroQol 5-Dimension.

	r	p
			Physical activity (non-hemodialysis days)		
Sedentary behavior (min)	-0.416	<0.001*
Prolonged sedentary bouts (≥5 min) (min)	-0.390	<0.001*
Prolonged sedentary bouts (≥10 min) (min)	-0.382	<0.001*
Prolonged sedentary bouts (≥30 min) (min)	-0.355	<0.001*
Prolonged sedentary bouts (≥60 min) (min)	-0.342	<0.001*
Median sedentary bouts (min)	-0.228	0.017*
Maximum sedentary bouts (min)	-0.436	<0.001*
Sedentary bouts (bouts)	0.054	0.576
Prolonged sedentary bouts (≥5 min) (bouts)	-0.194	0.043*
Prolonged sedentary bouts (≥10 min) (bouts)	-0.274	0.004*
Prolonged sedentary bouts (≥30 min) (bouts)	-0.313	0.001*
Prolonged sedentary bouts (≥60 min) (bouts)	-0.318	<0.001*
Sedentary bouts (min/bouts)	-0.271	0.004*
Prolonged sedentary bouts (≥5 min) (min/bout)	-0.228	0.017*
Prolonged sedentary bouts (≥10 min) (min/bout)	-0.242	0.011*
Prolonged sedentary bouts (≥30 min) (min/bout)	-0.284	0.003*
Prolonged sedentary bouts (≥60 min) (min/bout)	-0.301	0.001*

The relationships between various parameters of prolonged sedentary bouts and EQ-5D scores after adjustments for confounding factors were investigated by a multiple correlation analysis (Tables 3-5). Maximum sedentary bouts (minutes) (β = -0.389, p = 0.001) and sedentary behavior (minutes) (β = -0.377, p = 0.001) were identified as factors affecting HRQOL after adjustments for sex, the duration of hemodialysis, age, a history of diabetes mellitus, BMI, and albumin. Furthermore, the adjusted coefficients of determination (R²) of these parameters were higher than those of other parameters.

**Table 3:** Relationships between EQ-5D scores and sedentary bout parameters (min) on non-hemodialysis days by a multiple regression analysis *p < 0.05. VIF: variance inflation factor, EQ-5D: EuroQol 5-Dimension.

	β	ｐ	VIF	
					Dependent variable: EQ-5D scores				
Independent variables				
Sedentary behavior (min)	-0.389	0.001*	1.319	Adjusted R² = 0.199, p = 0.001*
Sex (men/women)	0.166	0.061	1.050	
Duration of hemodialysis (months)	-0.137	0.147	1.191	
Age (years)	-0.098	0.298	1.193	
History of diabetes mellitus	-0.012	0.893	1.135	
BMI (kg/m^2^)	0.047	0.623	1.232	
Albumin (g/mL)	0.094	0.306	1.139	
Dependent variable: EQ-5D scores				
Independent variables				
Prolonged sedentary bouts (≥5 min) (min)	-0.355	0.001*	1.308	Adjusted R² = 0.180, p = 0.001*
Sex (men/women)	0.172	0.056	1.058	
Duration of hemodialysis (months)	-0.151	0.112	1.179	
Age (years)	-0.116	0.219	1.175	
History of diabetes mellitus	0.005	0.955	1.119	
BMI (kg/m^2^)	0.033	0.735	1.220	
Albumin (g/mL)	0.081	0.388	1.162	
Dependent variable: EQ-5D scores				
Independent variables				
Prolonged sedentary bouts (≥10 min) (min)	-0.346	0.001*	1.304	Adjusted R² = 0.175, p = 0.001*
Sex (men/women)	0.175	0.054	1.061	
Duration of hemodialysis (months)	-0.156	0.100	1.174	
Age (years)	-0.117	0.218	1.179	
History of diabetes mellitus	0.009	0.920	1.115	
BMI (kg/m^2^)	0.026	0.785	1.213	
Albumin (g/mL)	0.086	0.363	1.158	
Dependent variable: EQ-5D scores				
Independent variables				
Prolonged sedentary bouts (≥30 min) (min)	-0.309	0.003*	1.297	Adjusted R² = 0.156, p = 0.001*
Sex (men/women)	0.126	0.060	1.187	
Duration of hemodialysis (months)	-0.165	0.085	1.171	
Age (years)	-0.126	0.193	1.187	
History of diabetes mellitus	0.022	0.808	1.106	
BMI (kg/m^2^)	0.010	0.919	1.199	
Albumin (g/mL)	0.096	0.313	1.152	
Dependent variable: EQ-5D scores				
Independent variables				
Prolonged sedentary bouts (≥60 min) (min)	-0.278	0.006*	1.270	Adjusted R² = 0.142, p = 0.002*
Sex (men/women)	0.159	0.084	1.052	
Duration of hemodialysis (months)	-0.162	0.097	1.186	
Age (years)	-0.143	0.138	1.164	
History of diabetes mellitus	0.036	0.697	1.091	
BMI (kg/m^2^)	0.014	0.886	1.213	
Albumin (g/mL)	0.094	0.328	1.164	
Dependent variable: EQ-5D scores				
Independent variables				
Median sedentary bouts (min)	-0.189	0.053	1.138	Adjusted R² = 0.110, p = 0.001*
Sex (men/women)	0.156	0.097	1.061	
Duration of hemodialysis (months)	-0.200	0.041*	1.143	
Age (years)	-0.197	0.039*	1.077	
History of diabetes mellitus	0.042	0.658	1.113	
BMI (kg/m^2^)	-0.033	0.736	1.168	
Albumin (g/mL)	0.131	0.174	1.124	
Dependent variable: EQ-5D scores				
Independent variables				
Maximum sedentary bouts (min)	-0.377	0.001*	1.188	Adjusted R² = 0.204, p = 0.001*
Sex (men/women)	0.117	0.184	1.040	
Duration of hemodialysis (months)	-0.137	0.144	1.188	
Age (years)	-0.163	0.070	1.082	
History of diabetes mellitus	0.047	0.594	1.063	
BMI (kg/m^2^)	0.598	0.598	1.234	
Albumin (g/mL)	0.627	0.627	1.204	

**Table 4:** Relationships between EQ-5D scores and sedentary bout parameters (bouts) on non-hemodialysis days by a multiple regression analysis *p < 0.05. VIF: variance inflation factor, EQ-5D: EuroQol 5-Dimension.

	β	ｐ	VIF	
					Dependent variable: EQ-5D scores				
Independent variables				
Sedentary bouts (bouts)	0.036	0.706	1.070	Adjusted R² = 0.078, p = 0.031*
Sex (men/women)	0.136	0.155	1.073	
Duration of hemodialysis (months)	-0.219	0.027*	1.130	
Age (years)	-0.222	0.022*	1.074	
History of diabetes mellitus	0.086	0.363	1.050	
BMI (kg/m^2^)	-0.004	0.679	1.170	
Albumin (g/mL)	0.152	0.121	1.112	
Dependent variable: EQ-5D scores				
Independent variables				
Prolonged sedentary bouts (≥5 min) (bouts)	-0.159	0.092	1.060	Adjusted R² = 0.102, p = 0.011*
Sex (men/women)	0.127	0.173	1.039	
Duration of hemodialysis (months)	-0.203	0.039*	1.143	
Age (years)	-0.219	0.021*	1.050	
History of diabetes mellitus	0.061	0.521	1.078	
BMI (kg/m^2^)	-0.011	0.908	1.199	
Albumin (g/mL)	0.135	0.163	1.122	
Dependent variable: EQ-5D scores				
Independent variables				
Prolonged sedentary bouts (≥10 min) (bouts)	-0.235	0.015*	1.125	Adjusted R² = 0.129, p = 0.003*
Sex (men/women)	0.147	0.111	1.045	
Duration of hemodialysis (months)	-0.196	0.043*	1.142	
Age (years)	-0.183	0.052	1.087	
History of diabetes mellitus	-0.038	0.683	1.097	
BMI (kg/m^2^)	-0.235	0.995	1.125	
Albumin (g/mL)	0.126	0.186	1.124	
Dependent variable: EQ-5D scores				
Independent variables				
Prolonged sedentary bouts (≥30 min) (bouts)	-0.284	0.006*	1.303	Adjusted R² = 0.143, p = 0.002*
Sex (men/women)	0.178	0.056	1.076	
Duration of hemodialysis (months)	-0.187	0.052	1.148	
Age (years)	-0.129	0.188	1.204	
History of diabetes mellitus	0.019	0.842	1.124	
BMI (kg/m^2^)	-0.008	0.935	1.183	
Albumin (g/mL)	0.124	0.191	1.123	
Dependent variable: EQ-5D scores				
Independent variables				
Prolonged sedentary bouts (≥60 min) (bouts)	-0.261	0.014*	1.349	Adjusted R² = 0.131, p = 0.003*
Sex (men/women)	0.170	0.069	1.070	
Duration of hemodialysis (months)	-0.166	0.090	1.187	
Age (years)	-0.134	0.178	1.221	
History of diabetes mellitus	-0.030	0.753	1.114	
BMI (kg/m^2^)	0.005	0.959	1.206	
Albumin (g/mL)	0.110	0.252	1.144	

**Table 5:** Relationships between EQ-5D scores and sedentary bout parameters (min/bouts) on non-hemodialysis days by a multiple regression analysis *p < 0.05. VIF: variance inflation factor, EQ-5D: EuroQol 5-Dimension.

	β	ｐ	VIF	
					Dependent variable: EQ-5D scores				
Independent variables				
Sedentary bouts (min/bout)	-0.224	0.024*	1.189	Adjusted R² = 0.122, p = 0.005*
Sex (men/women)	0.164	0.080	1.067	
Duration of hemodialysis (months)	-0.194	0.046*	1.146	
Age (years)	-0.173	0.071	1.117	
History of diabetes mellitus	0.041	0.662	1.099	
BMI (kg/m^2^)	-0.019	0.843	1.176	
Albumin (g/mL)	0.118	0.220	1.137	
Dependent variable: EQ-5D scores				
Independent variables				
Prolonged sedentary bouts (≥5 min) (min/bout)	-0.168	0.085	1.135	Adjusted R² = 0.103, p = 0.011*
Sex (men/women)	0.148	0.114	1.052	
Duration of hemodialysis (months)	-0.207	0.035*	1.137	
Age (years)	-0.187	0.053	1.111	
History of diabetes mellitus	0.066	0.485	1.068	
BMI (kg/m^2^)	-0.026	0.792	1.174	
Albumin (g/mL)	0.126	0.197	1.137	
Dependent variable: EQ-5D scores				
Independent variables				
Prolonged sedentary bouts (≥10 min) (min/bout)	-0.174	0.073	1.116	Adjusted R² = 0.106, p = 0.010*
Sex (men/women)	0.145	0.122	1.047	
Duration of hemodialysis (months)	-0.199	0.043*	1.147	
Age (years)	-0.189	0.050	1.102	
History of diabetes mellitus	0.071	0.451	1.060	
BMI (kg/m^2^)	-0.023	0.815	1.177	
Albumin (g/mL)	0.124	0.203	1.138	
Dependent variable: EQ-5D scores				
Independent variables				
Prolonged sedentary bouts (≥30 min) (min/bout)	-0.210	0.029*	1.117	Adjusted R² = 0.119, p = 0.005*
Sex (men/women)	0.135	0.145	1.039	
Duration of hemodialysis (months)	-0.168	0.090	1.197	
Age (years)	-0.198	0.035*	1.102	
History of diabetes mellitus	0.071	0.376	1.068	
BMI (kg/m^2^)	-0.001	0.992	1.204	
Albumin (g/mL)	0.114	0.241	1.146	
Dependent variable: EQ-5D scores				
Independent variables				
Prolonged sedentary bouts (≥60 min) (min/bout)	-0.242	0.017*	1.242	Adjusted R² = 0.127, p = 0.004*
Sex (men/women)	0.164	0.079	1.063	
Duration of hemodialysis (months)	-0.175	0.074	1.173	
Age (years)	-0.157	0.105	1.152	
History of diabetes mellitus	0.050	0.589	1.078	
BMI (kg/m^2^)	0.009	0.928	1.216	
Albumin (g/mL)	0.108	0.264	1.150	

## Discussion

The present study investigated the relationships between various parameters of prolonged sedentary bouts on non-hemodialysis days and HRQOL in patients on chronic hemodialysis. Sedentary behavior (minutes) and maximum sedentary bouts (minutes) correlated with HRQOL.

Patients on chronic hemodialysis must lie in a supine position for at least four hours three times a week. Tamura et al. reported that physical activity markedly and continuously decreased after the initiation of dialysis in patients with chronic kidney disease [15]. Johansen et al. showed that physical activity levels were significantly lower (by 30%) in patients on chronic hemodialysis than in healthy controls [16]. Therefore, exercise therapy, such as increasing physical activity, is important and is recommended for patients on chronic hemodialysis [17]. Since hemodialysis causes general fatigue, including body pain and sleep disturbance [18], exercise, such as increasing physical activity, is more effective on non-hemodialysis days than on hemodialysis days [19]. However, many patients on chronic hemodialysis are elderly, and their cardio-pulmonary function and muscle strength are significantly lower than those of healthy subjects [20]. Therefore, it is more difficult for elderly patients to increase their physical activity on non-hemodialysis days. We previously demonstrated that sedentary behavior (%) [9] and prolonged sedentary bouts (≥30 and ≥60 minutes; % and bouts) [11] correlated with HRQOL in patients on chronic hemodialysis. In the present study, we focused on various parameters of prolonged sedentary bouts in chronic hemodialysis patients on non-hemodialysis days to identify those affecting HRQOL. The results obtained showed that sedentary behavior (minutes) and maximum sedentary bouts (minutes), which were quantitative parameters, rather than other parameters, which were qualitative, were important factors affecting HRQOL.

Strategies to reduce quantitative parameters in patients on chronic hemodialysis, such as sedentary behavior (minutes) and maximum sedentary bouts (minutes), have yet to be established. Owari et al. reported reductions in sedentary behavior in community dwelling elderly Japanese subjects by using the “Active Guide” brochure published by the Ministry of Health, Labour and Welfare, Japan (2013) and additional documents related to the benefits of reducing sedentary behavior [21]. King et al. showed that in community dwelling subjects >45 years who sat for more than 10 hours a day, self-monitoring using a smartphone app for eight weeks reduced sedentary time spent sitting in front of the TV by 29.1 ± 84.5 min/day [22]. Ball et al. conducted a four-month intervention study in which non-monetary rewards were offered for behaviors that reduced sedentary behavior in inactive community residents aged 40-65 years, which decreased sitting times by 3.1 hours/day [23]. These studies did not include patients on chronic hemodialysis. Although difficulties are associated with reducing sedentary behavior, it may be possible in patients on chronic hemodialysis in clinical practice with an external stimulation and self-monitoring by others. In addition, the risk of falling is three-fold higher in chronic hemodialysis patients who are frail, regardless of age [24]; therefore, careful interventions are needed.

There are several limitations that need to be addressed. This was a cross-sectional, not a longitudinal study. Therefore, we were unable to prove the relationships between various parameters of prolonged sedentary bouts and HRQOL in the long term. Furthermore, enrolled patients on chronic hemodialysis were from one hospital in Onomichi, Japan, and the results obtained from this study cannot be applied to all patients on chronic hemodialysis in Japan. Moreover, the mechanisms underlying the effects of sedentary behavior (minutes) and maximum sedentary bouts (minutes) on HRQOL remain unclear. Hamilton et al. showed that sedentary behavior increased the risk of diabetes mellitus, hypertension, and all-cause mortality through glucose and lipid metabolism [25]. Nevertheless, reductions in sedentary behavior (minutes) and maximum sedentary bouts (minutes), which are quantitative parameters, may be beneficial for improving HRQOL in patients on chronic hemodialysis. A large-scale prospective study is needed to confirm this relationship.

## Conclusions

The findings from this cross-sectional study revealed a significant correlation between sedentary behavior, measured in minutes, and the duration of maximum sedentary bouts, also measured in minutes, with HRQOL in Japanese patients undergoing chronic hemodialysis. Specifically, both of these quantitative parameters of sedentary behavior were found to be associated with variations in HRQOL. This suggests that HRQOL appears to be more closely related to extended periods of continuous sedentary behavior, rather than being influenced by the effects of fragmented or segmented periods of sedentary time. Consequently, it can be inferred that implementing strategies to reduce the overall amount of sedentary time may lead to a positive improvement in HRQOL for these patients.
